# A bottom-up approach to assess verbal therapeutic techniques. Development of the Psychodynamic Interventions List (PIL)

**DOI:** 10.1371/journal.pone.0182949

**Published:** 2017-08-24

**Authors:** Antje Gumz, Karolin Neubauer, Julia Katharina Horstkotte, Michael Geyer, Bernd Löwe, Alexandra M. Murray, Denise Kästner

**Affiliations:** 1 Berlin University of Psychology (PHB), Berlin, Germany; 2 Department of Psychosomatic Medicine and Psychotherapy, University Medical Center, Hamburg-Eppendorf and Schön Klinik Hamburg Eilbek, Hamburg, Germany; 3 Department of Medical Psychology and Medical Sociology, University of Leipzig, Leipzig, Germany; 4 Academy for Psychotherapy Erfurt, Erfurt, Germany; Georgetown University Medical Center, UNITED STATES

## Abstract

**Objective:**

Knowing which specific verbal techniques “good” therapists use in their daily work is important for training and evaluation purposes. In order to systematize what is being practiced in the field, our aim was to empirically identify verbal techniques applied in psychodynamic sessions and to differentiate them according to their basic semantic features using a bottom-up, qualitative approach.

**Method:**

Mixed-Method-Design: In a comprehensive qualitative study, types of techniques were identified at the level of utterances based on transcribed psychodynamic therapy sessions using Qualitative Content Analysis (4211 utterances). The definitions of the identified categories were successively refined and modified until saturation was achieved. In a subsequent quantitative study, inter-rater reliability was assessed both at the level of utterances (*n* = 8717) and at the session level (*n* = 38). The convergent validity of the categories was investigated by analyzing associations with the Interpretive and Supportive Technique Scale (ISTS).

**Results:**

The inductive approach resulted in a classification system with 37 categories (Psychodynamic Interventions List, PIL). According to their semantic content, the categories can be allocated to three dimensions: *form* (24 categories), *thematic content* (9) and *temporal focus* (4). Most categories showed good or excellent inter-rater reliability and expected associations with the ISTS were predominantly confirmed. The rare use of the residual category “Other” suggests that the identified categories might comprehensively describe the breadth of applied techniques.

**Conclusions:**

The atheoretical orientation and the clear focus on overt linguistic features should enable the PIL to be used without intensive training or prior theoretical knowledge. The PIL can be used to investigate the links between verbal techniques derived from practice and micro-outcomes (at the session level) as well as the overall therapeutic outcomes. This approach might enable us to determine to what extent the outcome of therapy is due to unintended or non-theoretically relevant techniques.

## Introduction

Verbal techniques are the cornerstone of most psychotherapeutic methods. Language is one of the most important “tools” of the therapist, and it is the basic medium through which information is conveyed. [1] Consequently, empirical knowledge regarding the question of what kind of verbal techniques a therapist should or should not apply in his or her therapeutic work is of major practical importance. Bergin and Strupp [2] stated that research should play a significant role precisely in this area–through the application of systematic and increasingly refined comparisons between different techniques and the assessment of their differential effects. As an integral part of comparative therapeutic studies, it is crucial to differentiate therapists’ behaviors to be able to specify the extent to which techniques actually differ and how this ultimately affects treatment outcome. [2–4]

Within psychotherapy research, various measures have been developed to assess characteristics of verbal therapeutic techniques. [5] The existing measures have been developed to address various research questions such as assessing adherence and competence of the therapist regarding specific therapeutic orientations, distinguishing between therapeutic orientations, investigating microprocesses in psychotherapy, or analyzing the relationship between techniques and therapy outcome.

Some of the measures assess all possible verbal techniques, whereas others focus on selected theoretically important constructs such as “interpretation”. The focus is partly limited to only one or two aspects of verbal techniques (e.g. differentiation between “interpretation” vs. “non-interpretation” or “supportive techniques” vs. “interpretative techniques”).

**Fig 1:**
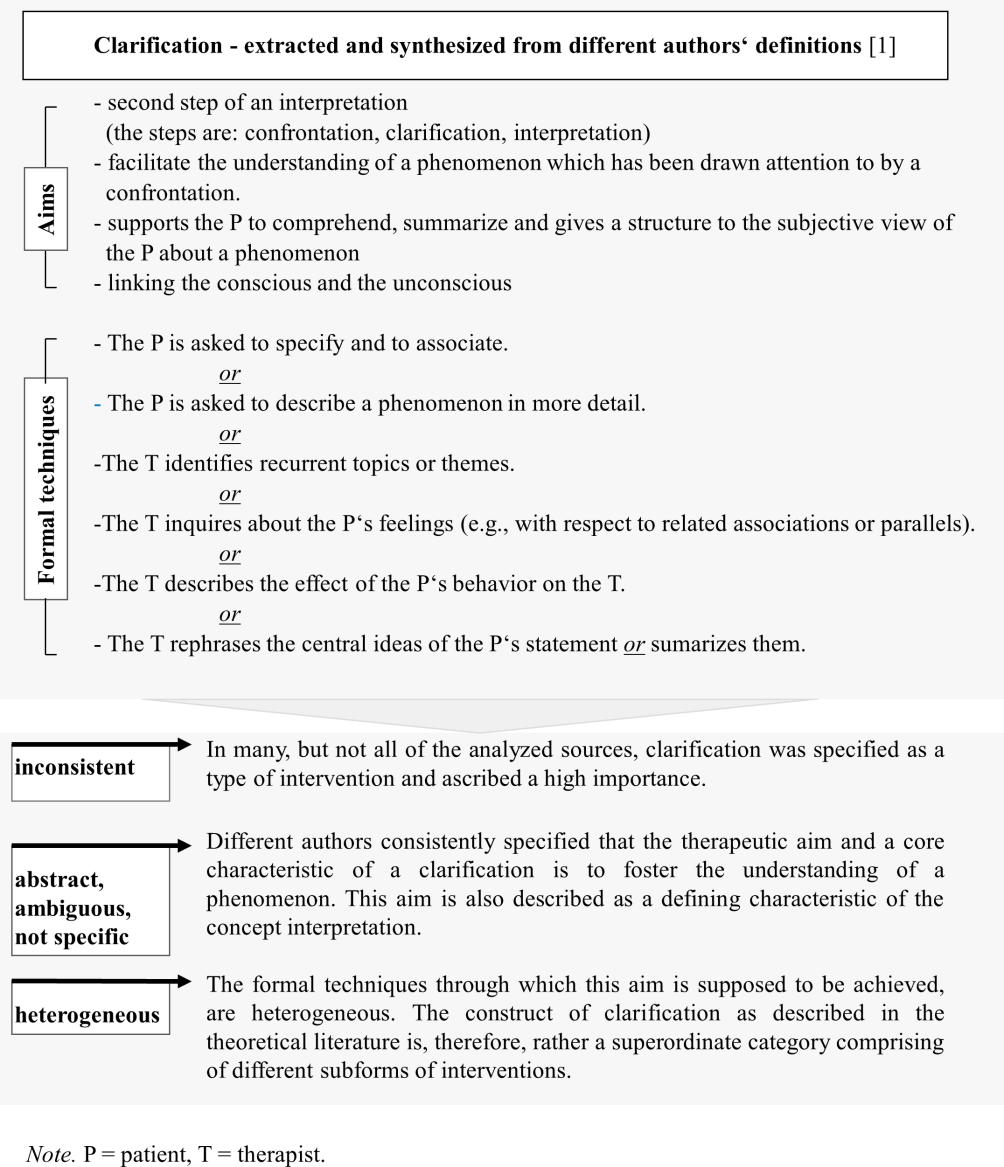
Example: Different definitions of clarification.

The majority of the assessment tools have used a top-down, theoretical approach to generate items. To our knowledge, there is no instrument which has yet been developed using a primarily inductive, bottom-up procedure comprising analyses of therapists’ utterances and audio- or videotaped therapy sessions to systematically describe and classify verbal therapeutic interventions. That means the measures currently in use have primarily been developed based on theoretical considerations, therapy manuals, literature research, clinical experience and expert discussion as well as previously existing scales. [5] As a result of this, they are afflicted with some difficulties which are inherent to the theoretical concepts, such as complexity, abstractness and ambiguity [6]. Fig 1 demonstrates this based on the example of the intervention type “clarification”. Different authors consistently specified that the therapeutic aim and core characteristic of a clarification is to foster the understanding of a phenomenon. This aim is also described as a core characteristic of the concept interpretation. The formal techniques through which this aim is supposed to be achieved, are heterogeneous. [1] Likewise, the definitions of the psychodynamic techniques interpretation and transference interpretation used in empirical studies are heterogeneous. [7–10]

Another aspect meriting discussion derives from the fact that top-down approaches do not allow us to investigate to what extent the outcome is due to unintended or even nontheoretically relevant techniques. [3] We do not know how specific verbal techniques actually are. It was shown that there appear to be differences between what therapists actually do in sessions and what they might be expected to do based on their theoretical background [11,12]. It would be useful to describe what therapists do without any reference to a specific theory. For this purpose, bottom-up, qualitative approaches could be helpful to develop scales in order to be able to gather theoretically unbiased, non-predetermined and comprehensive information. [13]

Furthermore, many items or subscales of the available measures simultaneously assess different characteristics of therapeutic techniques (e.g., “Make noninterpretive interventions, e.g., reflections, questions, provisions of information, clarifications, and confrontations”, ISTS [14]; “Therapist encourages the patient's expression and/or exploration of feelings in relation to a significant other (including therapist“, VTSS [15]). This prevents the separate evaluation of these features and their respective impact on treatment outcomes.

### Aims of the present study

Against this background we decided to develop a new approach to measure techniques. We were guided by two key principles: Firstly we felt it is useful to empirically identify verbal techniques applied in psychodynamic sessions using a bottom-up, qualitative approach in order to systematize what is being practiced in the field. Secondly, we wished to separate the basic features of verbal techniques into different dimensions in order to enable their separate evaluation. Thereby, we wanted to focus on directly observable features and to neglect latent characteristics whose assessment requires a higher degree of subjective inference. [5] Directly observable features relate to the semantic content (“what is said”) of therapeutic utterances. Latent features characterize the implicit pragmatic content of the utterances (“what is implicated” or “what is meant”, e.g., therapist’s intentions, therapeutic attitudes or qualitative characteristics like therapist’s empathy or competence). [5] Regarding the directly observable features, we differentiated three basic characteristics: 1. Form (i.e., the formal and structural manner in which the therapist responds to the patient’s experience, behavior or statement), 2. Temporal focus (i.e., the period of time to which the intervention refers and 3. Thematic content (i.e., the topic of the intervention).

Based on these aims we conducted two separate studies: In an extensive qualitative study, we identified the intervention categories resulting in the Psychodynamic Interventions List (PIL). To warrant the use of PIL in scientific research, we investigated the inter-rater reliability and convergent validity of the categories in an additional subsequent quantitative study.

## Qualitative study

## Method

### Approach

The categories of the PIL were developed based on transcripts of psychodynamic therapy sessions using Qualitative Content Analysis [13,16]. The main focus of this inductive approach was to proceed iteratively and to exclude latent constructs as much as possible. The aim was to achieve high agreement among different raters in the classification of all verbal interventions (typifying structuring). The level of analysis was defined as one therapist’s utterance, defined by the change of speaker with the patient). In some cases, two or more consecutive utterances of the therapist are summarized as one intervention according to concrete rules (Rules for Summarizing are presented in “S1 Text”), e.g., simultaneity: The patient talks simultaneously to the therapist, and the therapist continues to speak (interruption).

The initial codes were derived from the repeated analysis of the first transcript and group discussions. All of these initial categories were subject to modification in the subsequent process (eliminated, divided, combined, renamed, refined in their definition).

### Therapy sessions and participants

All psychotherapists of the Clinic for Psychosomatic Medicine and Psychotherapy of the University Hospital Leipzig were asked for study participation. Of 10 therapists 8 agreed to participate in the study with a minimum of one therapy. Upon confirmation newly admitted patients of the therapists were consecutively included: 13 patients were asked to participate, 3 declined, 10 agreed to participate. We aimed to capture a wide scope of verbal interventions and included sessions with (a) therapists of varying background and clinical experience (5 women, 3 men; 32–64 years; working experience: 1.5–38 years; professional background: 3 psychologists, 5 medical specialists; therapeutic background: 3 psychoanalytic psychotherapists, 5 psychodynamic psychotherapists), (b) varying characteristics and diagnoses of the patients (8 female, 2 male; 20–45 years; primary diagnoses: 1 depressive disorder, 3 anxiety disorders, 1 obsessive-compulsive disorder, 2 eating disorders, 1 somatoform disorder, 1 emotionally unstable personality disorder, 1 dependent personality disorder; percentage of comorbid personality disorders: 90%) and (c) individual therapy within different settings (4 outpatient, 6 inpatient). All therapies took place in the Clinic for Psychosomatic Medicine and Psychotherapy of the University Hospital Leipzig.

We decided to focus on more advanced phases of therapy at this stage of research. Video recording started mid-therapy course (seventh session) and was terminated before the final 7 to 10 sessions of therapy due to the naturalistic design of our approach. In so doing, patients had sufficient time to decide whether or not to participate in the study. Sessions were randomly selected from this pool of possible sessions.

In total, 4.211 utterances from 18 sessions were analysed. Participants agreed to the recording, transcription and analysis of the sessions and provided written informed consent. Procedures were approved by the University of Leipzig Ethics Commission.

### Procedure

The roles of therapists, transcribers, and researchers were separated. That means, therapists did not take part in the transcription, coding, or analyses of the sessions. The majority of the sessions were transcribed by a professional transcription service, a small proportion were transcribed by psychology graduate students who did not belong to the research group. The sessions were transcribed based on the approach of Mergenthaler [17].

All sessions were analyzed independently by two core researchers, who were involved in the entire coding process and attended all group discussions: one medical doctor with formal training in psychoanalysis and psychodynamic psychotherapy (AG, 15 years of clinical experience) and one psychologist (DK, 6 months of clinical experience). Additionally, the research group consisted of seven rotating members who coded various sessions and attended the respective group discussions (one medical doctor with 35 years of clinical experience, three psychology graduate students, three medical MD students). By combining a rotating team with two core members, we tried to avoid potential redundancies within the group discussion associated with an exclusively rotating approach as well as incorporating various points of view and new ideas of the additional team members. Furthermore, it is possible to test the comprehensibleness and the adequacy of the determined categories and definitions to that point with each newly involved team member [18]. Also, the rotating team with mainly naïve coders might have helped to minimize potential bias due to AG’s psychodynamic orientation. The focus on overt linguistic characteristics and the second core member (DK) might have also contributed to reduced bias. The open category “Other” was used for open coding, through which the addition of new categories was possible. The definitions and labeling of the categories were subsequently refined and modified. Utterances with total agreement were used as “typical” examples. The response format was also a subject of the group discussions as well as the categories themselves.

The generation of categories was terminated when the successive coding of three transcripts resulted in high agreement among the raters and there were no novel aspects that demanded the addition of a category (saturation).

## Results

### Identification of the PIL categories

The described inductive approach yielded a classification system with 37 categories. As part of the systematization process, the three separate characteristic dimensions *form* (24 categories), *temporal focus* (4 categories) and *thematic content* (9 categories) were differentiated. The *form* describes in which formal-structural manner the therapist verbally responds to the patient. The *temporal focus* refers to the period of time to which the intervention relates and the *thematic content* describes the topic.

**Fig 2:**
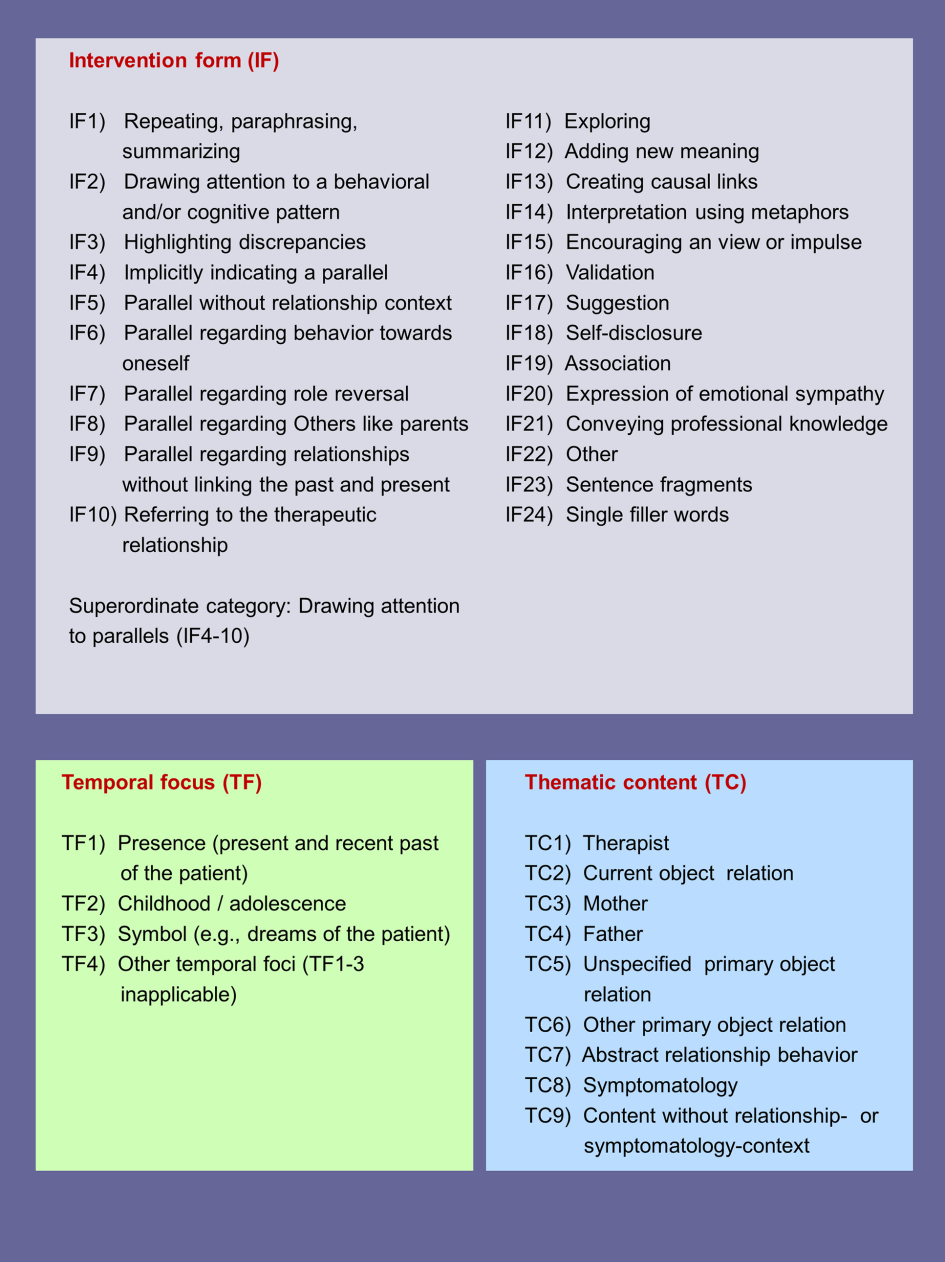
Categories of the Psychodynamic Interventions List (PIL).

**Fig 3:**
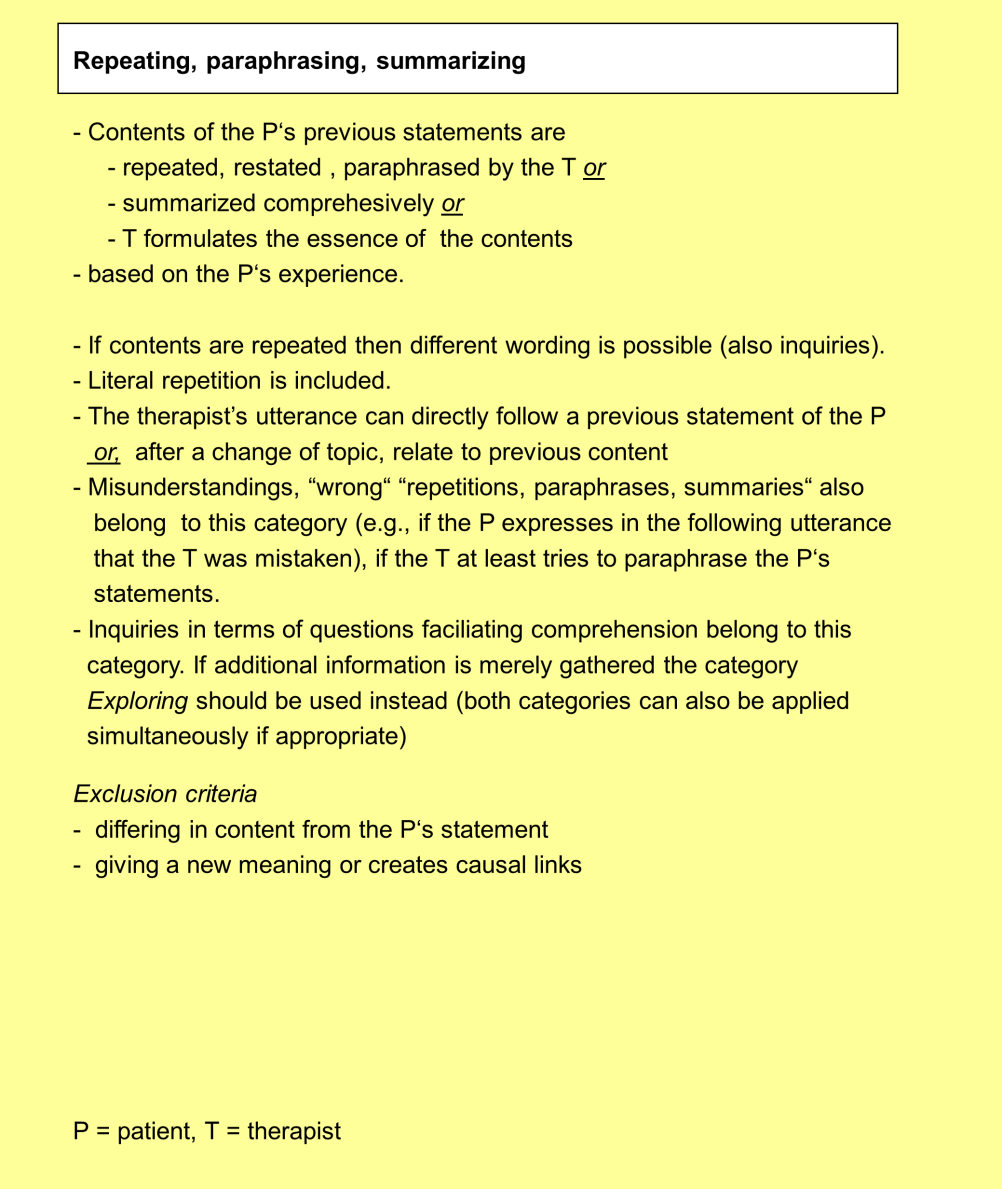
Definition of the category “Repeating, paraphrasing, summarizing”.

Fig 2 gives an overview of the labeling of all PIL categories. The short definitions of all categories are presented in “S1 Fig”. The much more detailed definitions of all categories including examples are specified in the Manual for the Psychodynamic Interventions List (PIL) (see “S2 Text”). Fig 3 shows the detailed definition of the category “Repeating, paraphrasing, summarizing” as an example. We decided to combine seven categories belonging to the formal dimension into a superordinate category “Drawing attention to parallels” due to their overlapping features as emerged from the qualitative analyses.

### Application of the PIL

The PIL may be used for both therapy training and research. For the application in scientific research, we tested and compared the appropriateness of both dichotomous and parametric ratings as well as the possibilities of single and multiple category ratings. The group discussion and first analyses showed that a substantial number of therapist utterances contained multiple categories. However, the relative importance of a certain category within a multiple category utterance was often judged to be unequal by the raters. Consequently, multiple coding and parametric rating appeared to be the best solution. The analysis of five randomly selected sessions and three different raters showed that between 44.0% and 56.8% of the therapists’ utterances contained multiple categories.

As a consequence, each utterance is rated for every category. Parametric ratings on the extent to which a category applies, ranging from “0 = the characteristics of the category do not apply” to “5 = the characteristics of the category completely apply” showed higher agreement among raters than dichotomous ratings.

The PIL ratings enable both analyses at the level of speech units (microanalytic coding, e.g., course of categories within a therapy session) and at the session level (global coding, percentage of applied categories in a complete session, e.g., contribution of a respective category to the experienced quality of the session). The percentage weighting can be calculated to examine the extent a particular category is applied in a session (dividing the sum of the values of the category of interest by the sum of the values of all categories in the respective dimension—form, temporal focus, thematic content).

The rating of one session takes three hours on average. The application requires prior training and profound knowledge of the PIL manual. Sample ratings of at least three sessions are recommendable for training purposes. The application of the PIL does not require clinical experience. For instance, medical students and students of psychology are fully suitable as raters when trained.

## Quantitative study

## Method

Aims of the quantitative study were to investigate (1) the inter-rater reliability and (2) the convergent validity of the PIL categories.

### Participants

#### Patients

Sixteen patients were included. Patients’ age ranged from 20 to 40 (*M* = 27.81, *SD* = 5.64), 12 participants were single (75%), twelve were employed (75%), two unemployed (12.5%) and two were still in higher education. Fifteen patients were female and received individual psychodynamic psychotherapy with 29 to 35 individual sessions. The sample also contained an individual long-term psychoanalytic psychotherapy with 220 sessions of a male patient. We decided to include this patient to increase the variability of our sample and because the sample was not used to investigate a clinical research question.

All patients terminated therapy without dropping-out. All patients suffered from a personality disorder with at least one comorbidity on Axis I, which were diagnosed based on the Structured Clinical Interview for DSM-IV Axis I and AXIS II Disorders [19,20], German: [21]. Exclusion criteria were bipolar affective disorders, psychotic disorders and acute suicidality. Seven patients were diagnosed with a dependent (43.75%), five with a narcissistic (31.25%), three with a borderline (18.75%) and one with an obsessive-compulsive personality disorder (6.25%). Comorbid diagnoses were anxiety disorders (*n* = 6, 37.50%), obsessive-compulsive-disorders (*n* = 1, 6.25%), eating disorders (*n* = 5, 31.25%), somatoform disorders (*n* = 3, 18.75%), depressive disorders (*n* = 12, 75.00%) and adjustment disorders (*n* = 1, 6.25%).

Patients provided written informed consent prior to commencing therapy. Study procedures were approved by the University of Leipzig Ethics Commission.

#### Therapists

The study included seven female and two male therapists each contributing 1–2 patients to the sample (age 32–45; five psychologists, four medical specialists). They were recruited within the University Hospital of Leipzig, Germany. Five had completed training in psychodynamic psychotherapy with at least five years of professional experience. Two had an additional qualification in psychoanalysis. The other four therapists were in advanced training of psychodynamic psychotherapy.

### Therapy sessions

In total, 345 50-minute psychodynamic and psychoanalytic sessions from 16 patients were video-taped. Recording started mid-therapy course (mean: seventh session) and was terminated before the final 7 to 10 sessions of therapy. First, we identified the two sessions with the intraindividual highest and the two sessions with the intraindividual lowest affiliation scores (as measured by the Intrex Questionnaire [22], see section “Measures”) per patient. We analysed one of the two 'high affiliation' sessions and one of the two 'low affiliation' sessions (which one of the two sessions was randomly chosen). In order to increase the number of sessions, six additional sessions from the session pool (three of the 'high affiliation' sessions and three of the two 'low affiliation' sessions) were randomly selected and included. This led to a total of 38 sessions.

Importantly, the sessions were restricted to be different to those selected for Study 1. That means sessions used in Study 2 did not overlap with those used for the development of the PIL (Study 1).

In order to describe the sessions more precisely, we compared the ISTS means as per the original ISTS publication [14] with the ISTS means of the PIL sessions (Table 1). The PIL sessions ranged between the means of the Interpretive Therapy sessions (STI) and the means of the Supportive Therapy sessions (SUP) with few exceptions which indicated a stronger emphasis towards the interpretative spectrum.

**Table 1:** Comparison of mean ratings for the items, subscales, and the full ISTS scale according to different treatment modalities and PIL sessions.

	STI	SUP	PIL sessions (n = 40)
**Supportive items**			
1. Gratify	0.36	2.91	0.98 (SD = 1.25)
3. Noninterpretive interventions	1.96	3.73	2.75 (SD = 0.83)
5. Guidance	0.19	2.38	0.60 (SD = 1.02)
7. Problem solving	0.07	1.55	0.55 (SD = 0.91)
9. Explanations	0.19	1.58	0.15 (SD = 0.59)
11. Praise	0.07	1.78	0.45 (SD = 0.79)
13. Personal information	0.15	1.56	0.98 (SD = 0.92)
**Interpretive items**			
2. Pressure	1.92	0.27	0.78 (SD = 1.07)
4. Uncomfortable emotions	2.31	0.52	2.30 (SD = 1.23)
6. Interpretations	3.31	0.57	3.15 (SD = 1.0)
8. Impression of therapist	1.34	0.02	1.58 (SD = 1.53)
10. Linking	1.17	0.02	1.18 (SD = 1.47)
12. Patient-therapist relationship	2.08	0.25	1.38 (SD = 1.45)
14. Impression of others	1.91	0.74	2.48 (SD = 1.41)
**Supportive subscale**	3.00	15.46	6.5 (SD = 3.69)
**Interpretive subscale**	14.04	2.40	12.83 (SD = 5.40)
**Full scale**	39.05	14.93	34.33 (SD = 8.34)

PIL = Psychodynamic Interventions List. STI = interpretive; SUP = supportive, Full scale: range: 0–56, 0–28: indicating supportive emphasis, 29–56: indicating interpretive emphasis

### Raters

Three female raters were involved in the psychometric evaluation, one being a medical doctor who developed the PIL as well as two independent psychology graduate students who were trained in applying the PIL. They were blind to any information about the patients, sessions and therapists.

### Measures

#### Interpretive and Supportive Technique Scale (ISTS) [23]

The ISTS was utilized to validate the PIL. The 14 items assess different interpretive and supportive interventions in dynamically oriented psychotherapies. Psychometric properties including inter-rater reliability have been shown to be at least satisfactory [23]. The ISTS was developed based on a treatment manual as well as clinical experience from its authors. It was developed with the goal to be widely applicable and to “be used by bachelor’s degree-level ratersˮ (p. 144). The interrater reliability was shown to be high among raters with this educational level [14]. Ratings were applied on a 5-point Likert-type scale after listening to a complete session [23,24].

#### Intrex Questionnaire [22]

This measure of interpersonal interactions from the patient’s perspective was the basis for the selection of sessions. We used the short form of the Intrex questionnaire [22] for multiple assessment of the therapeutic interaction. The intrex was developed on the basis of the “Structural Analysis of Social Behavior” model (SASB) [25]. The SASB is one of the most influential approaches to the categorization of interpersonal interactions and has been subject to considerable empirical examination and wide application [26]. Patients completed the questionnaire immediately after each therapy session. They each rated the therapeutic interaction on two foci of action (active and reactive behavior) as well as in two different directions (“How did I behave towards my therapist in today’s session?” and “How did my therapist behave towards me in today’s session?”; examples: “She unthinkingly ignored and neglected me.”, “I clearly and comfortably expressed my own thoughts and feelings to her.”). The items assess the interaction on a 10-point interval scale ranging from 0 (“never/not at all”) to 100 “always/completely”). We combined the Intrex scores of items 2 to 4 and 6 to 8 for each of the 2 directions and the active and reactive focus to form the weighted affiliation index as introduced by Gumz et. al. [27] and as proposed by Pincus et al. [28]. Pincus et al. showed that a weighted sum of the clusters had acceptable psychometric properties as well as good validity. A high affiliation index signifies the experience of affectionate interaction between the patient and therapist [29].

### Procedure

The roles of therapists, transcribers, and researchers were separated. That means, therapists did not take part in the transcription, coding, or analyses of the sessions. The majority of the sessions were transcribed by a professional transcription service, and a small proportion was transcribed by psychology graduate students who did not belong to the research group. The sessions were transcribed based on the approach of Mergenthaler [17]. Next, they were independently coded with the PIL by all raters. The ratings were conducted at the level of utterances of the therapist, defined by the change of speaker with the patient. That means, every utterance was rated for every category. One rater applied the ISTS. A 4-week time interval passed between ratings of the PIL and the ISTS in order to avoid memory effects.

### Data analysis

#### Inter-rater reliability

The inter-rater reliability was analyzed both on session level (*n* = 38) and at the level of utterances (*n* = 8717). Shrout and Fleiss’ [30] conservative intraclass correlations coefficient (ICC) model 2 was applied [31] The inter-rater reliability was calculated for the individual categories as well as for the three dimensions *form*, *thematic content*, and *temporal focus* using the dimensions’ mean ICC (2,1). The averaging was performed on Fisher’s Z-transformed ICC coefficients, which were then back-transformed into ICC coefficients. Fleiss‘ guidelines [32] were applied to interpret the ICC indices: >75 = excellent agreement, .40-.75 = moderate to good agreement, < .40 = poor agreement. (Fleiss (1981) referred to kappa-coefficients, but indicated that this set of criteria can be equivalently applied to intraclass correlations if the mean differences between the raters are considered to be part of the error. This approach is followed in the ICC analysis (2,1) [27] applied here.) For a more specific differentiation, we interpreted results from .40 to .59 as moderate and results from .60 to .75 as good reliability.

#### Sensitivity analyses of the inter-rater reliability

Variability. Given that a low ICC can be due to the low variability of a category [31], the standard deviations (mean of the three raters) of all categories with less than good reliability were examined.

Influence of the psychoanalytic sessions. In order to examine if the reliability of the psychoanalytic sessions systematically differed from the overall reliability, the ICC coefficients were separately analyzed for the psychoanalytic sessions at the level of utterances.

#### Convergent validity

It was expected that not all items would accord with other instruments and that the degree of overlap would vary in its extent because the PIL was developed inductively. We choose the ISTS as validation tool because it assesses psychodynamic verbal interventions and has established psychometric properties [23]. We formed correlational hypotheses regarding the size and direction of the associations for items with similar content. There were no equivalent items in the ISTS for the four PIL items on temporal foci, two thematic contents and 14 forms. The hypotheses are shown together with the corresponding results. Their detailed rationale can be requested from the authors (example: Medium positive correlations were expected between ISTS item 6 **“**Make interpretations”—“reference to one or more dynamic components”—and the PIL categories “Adding new meaning”, “Creating causal links” and “Interpretation using metaphors”. Rationale: ISTS item 6 includes more latent characteristics; the PIL differentiates three separate categories which are directly observable).

The mean percentage weightings of the three raters were applied. Depending on the normality of the distribution of the items (Kolmogorow-Smirnow test), Pearson’s *r* or Spearman’s *ρ* were calculated in order to examine links between the PIL categories and the ISTS items. The correlation coefficients were interpreted based on Cohen [33]: *r* < .30 as a small, *r*≥.30 as a medium and *r*≥.50 as large effect. Given that correlations coefficients represent effect sizes themselves [34], we focused on the magnitude of the correlations in the validity analyses. *P*-values are additionally reported (two-tailed testing, α < .01 in order to balance the risks of a potentially inflated Type I error rate in multiple testing and drawing conclusions in exploratory research [35]).

## Results

### Inter-rater reliability

The mean of the ICC coefficients was .80 at the session level (range .13-.98) and .68 at the level of utterances (range .10-.96). All ICC coefficients are presented in Table 2.

**Table 2:** Intraclass correlations (ICC) for each PIL category in the three characteristic dimensions: form, thematic content, temporal focus.

PIL category	Percentage weighting (SD)	session level *N = 38 sessions*		level of utterances *N = 8717*	
		ICC	95% CI	ICC	99% CI
**Form**					
Repeating, paraphrasing, summarizing	19.18 (7.84)	.80	[.67, .89]	.70	[.68, .71]
Drawing attention to a behavioral and/or cognitive pattern	9.46 (3.61)	.52	[.28, .70]	.65	[.63, .66]
Highlighting discrepancies	3.33 (1.79)	.44^V^	[.12, .67]	.54^V^	[.52, .56]
Implicitly indicating a parallel	.67 (1.08)	.76	[.63, .86]	.64	[.63, .65]
Parallel without relationship context	.60 (.61)	.24^V^	[.06, .45]	.36^V^	[.35, .38]
Parallel regarding behavior towards oneself	.12 (.44)	.88	[.80, .93]	.58^V^	[.56, .59]
Parallel regarding role reversal	.14 (.44)	.84	[.72, .91]	.68	[.67, .69]
Parallel regarding others and significant carers	.63 (1.04)	.92	[.86, .95]	.70	[.68, .71]
Parallel regarding relationships	1.54 (1.24)	.65	[.49, .78]	.58^V^	[.57, .60]
Referring to the therapeutic relationship	1.60 (1.52)	.78	[.66, .87]	.65	[.64, .67]
Exploring	16.17 (5.84)	.90	[.84, .94]	.87	[.86, .87]
Adding new meaning	18.55 (4.78)	.43	[.14, .66]	.69	[.68, .70]
Creating causal links	2.22 (1.32)	.49^V^	[.29, .67]	.51^V^	[.50, .53]
Interpretation using metaphors	.60 (1.13)	.64	[.47, .77]	.53^V^	[.51, .54]
Encouraging a view or impulse	1.07 (.98)	.30^V^	[.10, .50]	.27^V^	[.25, .29]
Validation	2.03 (1.54)	.68	[.53, .81]	.58^V^	[.56, .59]
Suggestion	2.70 (2.68)	.72	[.57, .83]	.64	[.63, .65]
Self-disclosure	1.73 (2.03)	.68	[.52, .80]	.61	[.60, .63]
Association	1.89 (1.29)	.53^V^	[.30, .71]	.53^V^	[.51, .55]
Expression of emotional sympathy	.69 (.93)	.69	[.54, .81]	.51^V^	[.50, .53]
Conveying professional knowledge	1.50 (5.45)	.97	[.94, .98]	.76	[.75, .77]
Other	10.65 (6.63)	.71	[.50, .84]	.60	[.58, .62]
Sentence fragments	2.85 (2.04)			.96	[.96, .96]
Single filler words	*	.77	[.59, .87]	.72	[.71, .73]
***Mean form***		.72		.65	
Superordinate category Drawing attention to parallels	.76 (.91)	.78	[.65, .87]	.71	[.70, .72]
**Thematic content**					
Therapist	25.68 (24.95)	.87	[.80, .93]	.76	[.75, .77]
Current object	21.64 (23.30)	.90	[.84, .94]	.81	[.80, .81]
Mother	6.51 (9.69)	.98	[.97, .99]	.89	[.89, .90]
Father	4.34 (7.21)	.96	[.93, .98]	.84	[.84, .85]
Unspecified significant carer	.24 (.39)	.41^V^	[.21, .60]	.36	[.34, .37]
Other significant carer	3.84 (12.82)	.95	[.92, .97]	.82	[.81, .82]
Abstract relationship behavior	13.92 (12.95)	.74	[.51, .86]	.66	[.63, .68]
Symptomatology	6.65 (9.91)	.89	[.82, .94]	.81	[.80, .81]
Other content without relationship or symptomology context	17.18 (15.21)	.81	[.70, .89]	.65	[.63, .66]
***Mean thematic content***		.90		.76	
**Temporal focus**					
Present	91.61 (10.37)	.88	[.80, .93]	.69	[.68, .71]
Childhood / adolescence	6.75 (9.49)	.91	[.83, .95]	.78	[.77, .79]
Symbol	1.02 (3.65)	.85	[.76, .91]	.72	[.71, .73]
Other temporal foci	.62 (1.21)	.13^V^	[-.05, .35]	.10^V^	[.08, .11]
***Mean temporal focus***		.79		.62	

PIL = Psychodynamic Interventions List. ICC = Intraclass correlation coefficients (2,1) based on Shrout & Fleiss [30]. Classification = classification according to Fleiss [32]: >.75: excellent, .60-.75: good, .40-.59 moderate, < .40 poor. Percentage weighting = sum of the values of the category of interest divided by the sum of the values of all categories in the respective dimension across all sessions. SD = standard deviation. ^V^Reliability classified as less than ‘good’ which might be explained by small variance as the standard deviation was below-average when compared to the other categories of the same dimension.

*The percentage weighting of the category “Single filler words” was 43.34%. To better illustrate the distributions of interest this category was not included in the calculation of the percentage weightings of the other categories.

Form: On average, the dimension form had a good reliability. Some categories had a moderate coefficient (session level: 5 categories; level of utterances: 8 categories); two categories on each level had poor ICC coefficients.

Thematic content: The mean reliability was excellent. The reliability coefficients ranged between good and excellent with the exception of the category “Unspecified primary object relation” which yielded a moderate ICC at the session level and a poor ICC at the level of utterances. Importantly, this category only accounted for 0.24% of the percentage weightings of all thematic contents.

Temporal focus: On average, the temporal foci had excellent reliability at the session level and a good reliability at the level of utterances. The categories showed good or excellent reliability scores except the residual category “Other temporal foci” which had a poor ICC at both the utterance and session levels and accounted for 0.62% of the percentage weightings of all temporal foci.

### Sensitivity analyses

Variability. Seven of the nine categories which did not at least had a good reliability score at the session level had a low variance. At the level of utterances, this criterion applied for 11 of the 12 categories (Table 2).

Influence of the psychoanalytical sessions. The ICC coefficients which were calculated for the psychoanalytical sessions were also moderate to excellent (form: *M* = .58; range -.01-.96; thematic content: *M* = .99; range .44–1.00; temporal focus: *M* = .57; range -.01-.90).

### Convergent validity

Expectations regarding the size and direction of the correlations were generally confirmed (Table 3). Hypotheses 1b, 9 and 16 yielded higher correlation coefficients than expected, hypothesis 10b yielded a smaller correlation coefficient than expected, but was still significant. In total, 17 of the 21 correlations were significant, *p* < .01.

**Table 3:** Hypotheses and results for the correlations between the PIL categories and ISTS items.

Hypo-thesis	PIL category	ISTS items	Hypothesi-zed size of correlation	*r / ρ*
**Thematic contents**				
1 a	Therapist	Direct attention to the patient’s subjective impression of the therapist	*large positive*	*ρ* = .82***
1 b	Therapist	Make links between the patient’s relationship with the therapist and the patient’s relationships with others	*medium positiv*e	*ρ* = .67***
1 c	Therapist	Focus on patient and therapist in the treatment situation rather than patient and significant others outside treatment situation	*large positive*	*ρ* = .84***
2	mean of Current object relation; Mother; Father; Unspecified primary object relation; Other primary object relation; Abstract relationship behavior	Direct attention to the patient’s subjective impression of others outside the treatment situation	*large positiv*e	*ρ* = .70***
**Form**				
3	Parallel regarding Others like parents	Make links between the patient’s relationship with the therapist and the patient’s relationships with others	*medium positiv*e	*ρ* = .35
4	Parallel regarding rela-tionships without lin-king past and present	Make links between the patient’s relationship with the therapist and the patient’s relationships with others	*medium positiv*e	*ρ* = .41**
5 a	Referring to the therapeutic relationship	Direct attention to the patient’s subjective impression of the therapist	*large positive*	*ρ* = .71***
5 b	Referring to the therapeutic relationship	Make links between the patient’s relationship with the therapist and the patient’s relationships with others	*large positive*	*ρ* = .54***
5 c	Referring to the therapeutic relationship	Focus on the patient and therapist in the treatment situation rather than the patient and significant others outside the treatment situation	*large positive*	*ρ* = .68***
6	Adding new meaning	Make interpretations	*medium positiv*e	*ρ* = .48**
7	Creating causal links	Make interpretations	*medium positiv*e	*ρ* = .47**
8	Interpretation using metaphors	Make interpretations	*medium positiv*e	*ρ* = .36
10 a	Validation	Gratify the patient, i.e., make the patient feel good rather than anxious in the session	*large positiv*e	*ρ* = .55***
10 b	Validation	Praise the patient	*large positiv*e	*ρ* = .48**
11	Suggestion	Engage in problem-solving strategies with the patient, i.e., generating and evaluating alternative solutions to external life problems	*medium positiv*e	*ρ* = .43
12	Self-disclosure	Display personal information, opinions, and/or values	*large positiv*e	*ρ* = .53***
13	Expression of emotional sympathy	Gratify the patient, i.e., make the patient feel good rather than anxious in the session	*large positiv*e	*ρ* = .56***
16	Superordinate category “Drawing attention to parallels”	Interpretive subscale	*medium positive*	*r* = .53**

PIL = Psychodynamic Interventions List. ISTS = Interpretive and Supportive Technique Scale [24]. The detailed rationale for hypotheses can be requested from the authors.

**p < .01

***p < .001.

## Discussion

The main purpose of this work was to identify and systemize verbal psychodynamic techniques used in clinical practice. The categories were derived inductively based on transcripts of therapy sessions. The analysis revealed a classification system with 37 categories (Psychodynamic Interventions List, PIL) with precise definitions based on observable characteristics. According to the semantic content, they can be allocated to three dimensions: *form* (24 categories), *thematic content* (9) and *temporal focus* (4).

We investigated the inter-rater reliability and convergent validity of the categories in an additional subsequent quantitative study to warrant the use of the PIL in scientific research. Overall, the majority of the PIL categories have good or excellent reliability scores. Moreover, almost all categories which did not have at least ‘good’ reliability coefficients had a low variance, which might have influenced the results. With respect to convergent validity, the results showed that the correlational hypotheses were predominantly confirmed.

It should be noted that the characteristic features of our categories differ noticeably from theoretical categories. While the theoretical concepts are more complex and contain more abstract information, the newly developed categories are generally defined more explicitly and in more detail. For example, the category “Adding new meaning”, is, according to its newly derived empirical definition (see “S1 Fig”), not synonymous to an interpretation. The interventions belonging to the formal dimension are categorized in a more differentiated manner which is reflected by a larger number of categories.

Our inductive approach inevitably reduces information comprised in theoretical concepts. Psychodynamic concepts such as transference and resistance have a long tradition and have been refined by many authors [1]. They form the basis of many psychotherapeutic approaches ranging from traditional psychoanalysis to relatively new trends of the “third wave” such as schema therapy [36] and are, therefore, of great importance. Some aspects of the theoretical constructs “conflicts” and “resistance” can be found in the PIL category “Highlighting discrepancies”. In the PIL, the term “discrepancy” comprises a formal relationship between elements which can be identified in the therapist’s utterances without including theoretical concepts. Of other theoretically crucial constructs, different aspects are covered separately in the PIL, e.g., transference interpretation: The definitions of a transference interpretation used in empirical studies differ crucially. They only agree in the aspect that a transference interpretation must comprise of a reference to the therapist [37]. This aspect of a transference interpretation is covered by the thematic PIL category “therapist”. In the theoretical literature, transference is generally understood as a manifestation of unconscious object representations in the relationship with the therapist or, more generally, with respect to a current relationship. Based on this characterization, addressing the repetition of a relationship pattern can be understood as another facet of a transference interpretation (see [7]). This component is covered in the PIL with the category “Parallel regarding others and significant carers”. Another potential aspect of the concept transference interpretation can be identified through the PIL category “Adding new meaning”.

The strength of our approach lies in the fact that the measure emerged from a bottom-up, qualitative approach and that it has multiple dimensions. In contrast, most intervention assessment tools have used a top-down, theoretical approach to generate items. Furthermore, the clear focus on overt linguistic features might make the PIL applicable to a wide range of potential users without intensive training or theoretical knowledge. Finally, the rare use of the residual category “Other” implies a high degree of completeness of the identified interventions.

Barber [3] emphasized that we need more sophisticated empirical knowledge regarding the question which techniques really matter and which should be classified as „clinical lore”(p. 325). This includes the question to what extent the outcome is due to unintended or nontheoretically relevant techniques. [3] An optimal solution would be to define techniques without reference to therapeutic methods and to find clear unambiguous designations for specific techniques so that the same thing is not called by different names. [5] For this purpose, bottom-up, qualitative approaches are helpful to develop scales in order to gather non-predetermined and comprehensive information. In this sense, the bottom-up development of the PIL might make it particularly applicable to third-wave cognitive therapy treatments which incorporate techniques being similar to the psychodynamic approach, yet might use different language to describe those techniques, It would be interesting to compare the frequency of use of specific categories in cognitive behavioral and psychodynamic therapies in future studies.

It is worth noting that the authors of some of the existing measures (e.g., CPPS, [38], PIRS, [39] conducted regular rater discussions to prevent rater drift. One must consider that the application of these instruments is, strictly speaking, limited to the used number of raters in order to ensure the documented reliability. That gives a certain advantage to the application of the PIL which can be applied by one rater only but is otherwise time-consuming (approximately three hours per session).

An additional application of the PIL (besides for psychotherapy research purposes) is to use the instrument as a learning tool for therapy training. Due to the PIL’s atheoretical orientation and our bottom up approach that aims to comprehensively describe verbal interventions, the PIL could be a useful training aid, especially for novice therapists.

Several limitations need to be mentioned: Importantly, we only examined one aspect of validity. In particular, the external validity of the PIL needs to be investigated in the future. We applied the PIL to a relatively small sample of patients with personality disorders. However, it would be interesting to extend the use of the PIL to other patient groups as well as to specific psychodynamic approaches (e.g. mentalization-based therapy) or even other theoretical backgrounds (e.g. cognitive-behavioral therapy).

It is important to note that it is difficult to establish hypotheses to test the validity of the PIL because the inductive approach requires the development to be as unbiased as possible from theoretical assumptions. Accordingly, it is not possible to examine criterion validity by showing whether the categories reflect “true” therapeutic orientation. Although our categories are derived from psychodynamic sessions, we cannot assume that the particular categories are solely or more frequently used in psychodynamic therapy. Nevertheless, we are currently preparing an analysis on the basis of a larger sample size in which we are going to test associations between the PIL categories and the session outcome as well as the outcome at the end of therapy. These results will further contribute to the validation of the PIL.

We restricted our analyses to the level of component skills [40], i.e. techniques at the level of therapists’ single utterances at the smallest unit of measurement. With that, we specifically focused on therapists’ techniques. Patients’ utterances are not the focus of our investigation. Of course, as known from experience, the patient also contributes to the progress of therapy by performing interventions such as paraphrasing, adding new meaning, or creating causal links. It would surely be worthwhile for future research to investigate to what extent the PIL can also be applied to patients’ utterances. Moreover, we did not define an intervention to be a longer process in which patient and therapist are mutually involved. [41,42] Importantly, the PIL ratings enable both consideration of the pattern of interventions, e.g., course of categories within a therapy session, and analyses at the session level (percentage of applied categories in a complete session, e.g., contribution of a respective category to the experienced quality of the session). Furthermore, it would be interesting to assess the agreement of PIL ratings between therapists, patients and external raters. Within the current studies we did not analyze these different perspectives.

Another limitation is that we did not include the first few sessions and the termination of treatment. Thus, with respect to the completeness of the list, our results are limited to sessions in advanced stages of therapy. Possibly, additional categories could be identified in the first and last sessions of therapy by means of qualitative methods. Nevertheless, the PIL can be applied to all sessions, including the first and the last, by using the residual category “Other” for types of techniques which have not yet been specified. In this way, differences in the frequency of use of certain categories, depending on the stage of therapy would become apparent.

Future research should aim to have larger sample sizes. To this end, it would be useful to establish a larger practice research network in which psychotherapists and researchers collaborate [43]. The atheoretical orientation and the clear focus on overt linguistic features might make the PIL applicable to a wide range of potential users without intensive training or theoretical knowledge. The PIL can be used to investigate the links between verbal techniques derived from practice and the micro-outcome in the respective sessions (e.g. relationship variables, or other common factors), as well as the overall therapeutic outcome. Castonguay [4,43] named the investigation of the interaction between technique variables, participants, and relationship for different clinical disorders as one of the two most important directions of future research and a matter of great interest to clinicians.

## Supporting information

S1 Fig Short definitions of the categories of the Psychodynamic Interventions List (PIL). (PDF)

S1 Text Concrete rules for summarizing utterances of the therapist as one intervention. (PDF)

S2 Text Manual for the Psychodynamic Interventions List (PIL). Detailed definitions of all categories including examples.
(PDF)
