# Supplementary material for: A bottom-up approach to assess verbal therapeutic techniques. Development of the Psychodynamic Interventions List (PIL)
Source: PLoS One. 2017 Aug 24;12(8):e0182949. doi: 10.1371/journal.pone.0182949 (PMC5570429; doi:10.1371/journal.pone.0182949)
Supplement: S1 Fig — (PDF) [file pone.0182949.s001.pdf]

Short definitions of the Psychodynamic Interventions List (PIL) categories

Intervention form (IF; 24 categories)

IF1) Repeating, paraphrasing, summarizing
The contents of the P's previous statements are repeated, paraphrased, restated, differentiated, summarized or the essence is extracted.

IF2) Drawing attention to a behavioral and/or cognitive pattern
The T directs the P's attention to a phenomenon, provides the P with a tangible perception of the P‘s behavioral or cognitive pattern.

IF3) Highlighting discrepancies
Highlighting contrasting aspects or drawing attention to discrepancies or contradictions of thoughts, experiences or behaviors.

IF4) Implicitly indicating a parallel
The T implicitly indicates possible parallels between experiences without specifying the parallel (e.g., "Does this event remind you of something else?").

IF5) Parallel without relationship context*
The T indicates parallels between topics, thoughts, experiences, behaviors, which do not refer to a relationship.

IF6) Parallel regarding behavior towards oneself
The T indicates parallels between the P's behavior towards him- or herself and the manner others have behaved towards the P.

IF7) Parallel regarding role reversal
The T indicates parallels between the P's behavior towards others and the manner others have behaved towards the P.

IF8) Parallel regarding others like parents
The T indicates an association between a P's perception of the behavior of one or more current object relations (e.g., partner, therapist) and the P's experience of a significant carer (e.g., mother).

IF9) Parallel regarding relationships without linking the past and present
The T indicates parallels between typical current or past interpersonal behavioral pattern without linking past and present.

IF10) Referring to the therapeutic relationship
The T directly relates the P's statements to the therapeutic relationship.

IF11) Exploring
Gathering new information with or without a direct question.

IF12) Adding new meaning
The T adds a new meaning to the contents which are described by the P, links the contents to a not previously verbalized context or integrates them into a new context.

IF13) Creating causal links
The T makes causal links between thoughts, feelings or behaviors; the focus is the causality (e.g., "…because..").

IF14) Interpretation using metaphors
An allegory, narrative, proverb or aphorism is introduced by the T to make the P's experience more tangible. It has to add meaning which would have not developed without the metaphor; more than pure repetition and summary in figurative language.

IF15) Encouraging an view or impulse*
The T encourages a thought, view or behavior, which has previously been hinted at by the P, but the P had not dared to openly discuss it.

IF16) Validation

The T expresses encouragement, validates a view, perception, behavior or impulse of the P.

IF17) Suggestion

The T gives advice or a recommendation, proposes solutions or proposes his/her experience or asks the P to do or refrain from doing something.

IF18) Self-disclosure

The T shares his or her personal experience with the P. The T communicates the effect the P's statements and behaviors have on the T; for example, which feelings and reactions they trigger in the T or the T describes his/her own reactions to the P's statements and behaviors.

IF19) Association

The T recalls thematic content which was mentioned by the P in previous sessions or the T reveals pictures, memories, stories which occur to him or her.

IF20) Expression of emotional sympathy

Verbally expressed reaction to the P's statements (sympathy, comfort, emotional consolation with the P's experience).

IF21) Conveying professional knowledge

The T conveys professional knowledge, explains an issue or a fact; the T remains abstract, general, theoretical and neutral.

IF22) Other

Everything, which does not fulfill the criteria of the other categories, including organizational matters.

IF23) Sentence fragments

An utterance which is not completed in the following utterance and does not form a complete sentence.

IF24) Single filler words

e.g., "hmm", "nah".

IF4-IF10) Superordinate category Drawing attention to parallels

Suggestion of a common denominator that describes similarities between different statements, topics and experiences. The sub-categories describe variants with regard to content.

Thematic content (TC; 9 categories)

TC1) Therapist

TC2) Current object relation

current relationships, e.g., with partner, children, friends, colleagues

TC3) Mother

TC4) Father

TC5) Unspecified primary object relation*

TC6) Other primary object relation

→ **Primary object relation**

Significant others who have shaped the personality structure in the childhood / adolescence (the category is applied when these objects are referred to in the past or in the present)

TC7) Abstract relationship behavior

TC8) Symptomatology

Symptoms in the strict sense, e.g., panic attacks, depression, compulsions, eating disorders, pain

TC9) Content without relationship- or symptomatology-context

Temporal focus (TF; 4 categories)

TF1) Presence
Present and recent past of the patient (not including childhood and adolescence)
TF2) Childhood / adolescence
TF3) Symbol
A topic or theme is addressed by means of a dream, fairy tale, metaphor etc.
TF4) Other temporal foci (not including presence, childhood / adolescence or symbol)*

P=patient, T=therapist. The detailed definitions of the categories can be requested from the authors. *= four categories which yielded poor reliability coefficients and should be revised in future studies.
