# Supplementary material for: A bottom-up approach to assess verbal therapeutic techniques. Development of the Psychodynamic Interventions List (PIL)
Source: PLoS One. 2017 Aug 24;12(8):e0182949. doi: 10.1371/journal.pone.0182949 (PMC5570429; doi:10.1371/journal.pone.0182949)
Supplement: S1 Text — (PDF) [file pone.0182949.s002.pdf]

Rules for Summarizing

Two or more consecutive utterances of the therapist are summarized if certain conditions apply. If conditions named under 1. or 2. or 3. are met, therapeutic utterances have to be summarized. If they are not met, utterances are not summarized.

1. The patient speaks at the same time as the therapist but the therapist has the last word (interrupting the speaker)

- The transcript shows this as follows: A **plus (+)** is written prior to the word the therapist is saying that is interrupted by the patient. In addition, a plus is written after the word the patient is saying upon interrupting the therapist.
- The following examples demonstrate the use of the plus (+).
- The first example illustrates what it looks like when the therapist has the last word, while the patient speaks simultaneously. Here, the therapeutic utterances have to be summarized.

T/ You have an +inflammation of a vein
P/ yes yes.+
T/ that you simply cannot get rid of

The speaker, i.e. the therapist, has the last word. – summarize.

Counterexample:
T/ like this, right? and this? well, I don't really understand this, because I think +with your
P/ that this is+ relatively low
T/ yes, so it is – still? slightly above average

The interrupter, in this case the patient, has the last word. – Despite the plus, do not summarize (unless the conditions under 2. and 3. are met).

Exceptions (There are 3 exceptions to this first rule)

a) The patient interrupts the therapist (indicated by a "+"), but this is directly followed by a **pause** (indicated by "(p:duration of pause)").

T/ There certainly are mistakes that no one notices – maybe you don't even notice it as a mistake yourself – and there is no! assurance over whether it was a mistake +or not
P/ yes+(p:00:00:08) [1]
T/ i i resist the term 'mistake' - um --- you sort of uh thought of something else than - what was planned, right?

There is a pause between the patient interrupting and the therapist continuing to speak - do not summarize

b) The patient interrupts the therapist (indicated by a "+"), but this is followed by the **therapist uttering a filler word only**.

T/ although you were not actually there today +so in this sense
P/ nah but still+
T/ uh-huh

After the patient interrupts the therapist, only a filler word is uttered - do not summarize

[1] Please note: Pauses lasting 2 seconds or longer are being transcribed (Mergenthaler 1992)

c) The patient interrupts the therapist (indicated by a “+“), but this is followed by the therapist uttering a **filler word** and then a **pause** (indicated by “(p:duration of pause)”).

T/ are you assuming this do you know +this
P/ i am assuming yes+
T/ uh-huh (<P1>:00:00:06) but then you could in fact expect that the others understand? and that it

After the patient interrupts the therapist, a filler word is uttered and this is followed by a pause prior to the therapist speaking again - do not summarize.

Despite exceptions, summarizing is necessary if conditions named under 2. or 3. are met:

2. The therapists‘ individual utterances (i.e. speech units) are only meaningful when they are summarized, not by themselves.
(This also applies if one out of two consecutive utterances is meaningful on its own, but the other one is not.)

3. It is impossible to meaningfully allocate utterances to specific categories without summarizing them. Instead, “Other” or “Sentence Fragments” would have to be chosen.
By summarizing, the allocation to the categories becomes more meaningful and obvious. (This also applies if out of two consecutive utterances only one can be clearly allocated and the other one needs to be summarized.)
