# Supplementary material for: A bottom-up approach to assess verbal therapeutic techniques. Development of the Psychodynamic Interventions List (PIL)
Source: PLoS One. 2017 Aug 24;12(8):e0182949. doi: 10.1371/journal.pone.0182949 (PMC5570429; doi:10.1371/journal.pone.0182949)
Supplement: S2 Text — Detailed definitions of all categories including examples. (PDF) [file pone.0182949.s003.pdf]

Gumz, A. (2016).
Manual for the Psychodynamic Interventions List (PIL)

Dear Raters!

1. To begin with, please carefully read all category descriptions within this **rating list**. You might have to read each category description several times in order to understand the *semantic features* of each category and the differences between the categories. The category descriptions are structured like a checklist. To allocate a therapeutic utterance (i.e. speech unit) to a category, all category features have to apply. Please note whether the word *or* is written in italics in between two features – in this case, only one of the two features has to apply in order to fulfill a category.

   Using the **scale** below, please rank how strongly the features apply to each category.

2. *All listed features have to apply* in order to fulfill a category (if some but not all features apply, this would be ranked as "0").The metric allocation is meant to show how strongly, noticeably or obviously the features apply.

   0 not at all

   1 hardly, slightly, only a little

   2 somewhat, moderately

   3 fairly, considerably

   4 strongly, obviously

   5 completely, absolutely, clearly

3. Please adhere strictly to the text. Please rate from a purely formal perspective! Avoid interpreting or analyzing therapeutic utterances or incorporating something you "read between the lines" into your decision.

   Example: Frequently asked question: What do I do if the T says something which can be allocated to a certain category and is then interrupted by the P who completes the utterance? → Only rate what is there. Do not add what the P said to what the T started saying. Another example: "I see" is a filler word and not an "Expression of emotional sympathy", even if it appears (i.e. sounds*)* especially interested or involved.

   The boundaries between the categories are often fluid. If, for example, a therapeutic utterance was rated with a "5" for "Repeating, rephrasing and summarizing" and a "2" for "Interpreting", it would clearly be allocated to "Repeating, rephrasing and summarizing", while having some features of "Interpreting".

4. Every utterance (i.e., row in the Excel table) represents a cohesive rating unit. Please consider *all categories per speech unit*, even if you have an immediate idea which category would apply. It is possible that other categories might also apply. Example: T: "Could you clarify in more detail, why…", "You already mentioned before, that…" → Here, values could be given for "Exploration" as well as "Repeating, rephrasing and summarizing".

5. The category “Other“ is meant to rate how strongly the respective therapeutic utterance is characterized by features not fully captured by the other categories. If the therapeutic utterance can be captured well and comprehensively by other categories, this category would be rated as “0”. If the characteristic features of the therapeutic utterance cannot/ only to a small extent be captured by other categories, this category would be rated as “5”.
6. Examples within the rating list: An example is provided for each of the formal categories. The examples were taken from previously transcribed and rated sessions. Raters found all examples to apply “completely, absolutely, clearly” (“5”).

Thank you and enjoy rating!

Rating List

T = Therapist, P = Patient

Dimension 1 – Form (24 categories)

1. Repeating, paraphrasing, summarizing

Contents of the P‘s previous utterances are:

- Repeated, restated, paraphrased by the T *or*
- Summarized comprehensively; the T formulates the essence of the contents.
- Based on the P‘s experience.
- If contents are repeated, different wording is possible (also inquiries).
- Literal repetition is also included.
- “Repeating, paraphrasing, summarizing” can directly follow an utterance of the P *or,* after a change of topic, relate to previous content.
- Misunderstandings, “wrong“ “Repetitions, paraphrases, summaries“ also belong to this category (e.g., if the P expresses in the following utterance that the T was mistaken), if the T at least tries to understand the P‘s utterances.
- Inquiries/ questions that facilitate comprehension also belong to this category. If additional information is merely gathered, the category “Exploring” should be used instead (however, both categories can also apply simultaneously).

Exclusion criteria:

- If the T’s utterance differs in content from the P‘s utterance.
- If the T is giving a new meaning or is creating causal links.

Example:

T: why would you prefer defiance? if you could feel it („Exploring“, ranked as „5“)
P: yes because i would know better what to do with it
T: uh-huh – uh-huh
P: because apathy is so difficult – difficult to fix!?
T: +uh-huh
P: and +defiance! that’s an active reaction! so
T: uh-huh - so the apathy is almost worse at the moment **(“Repeating, paraphrasing, summarizing”, ranked as „5“)**
P: yes

2. Drawing attention to a behavioral and/or cognitive pattern

- The T directs the P's attention to a phenomenon.
- The T confronts the P with a tangible perception of the P's behavioral or cognitive patterns, shares own observations about the P's behavioral or cognitive patterns.
- Perceptions of the T have to specifically relate to observable behavior, situations or experiences and thought processes as described by the P (verifying perceptions).
- The T describes something tangible and thereby highlights it.
- The T remains neutral (Expressing interest, astonishment ("I find it interesting…", "What I find astonishing/ remarkable…") fulfills criteria for neutrality. → Exception: The emphasis of the remark is put on communicating the T's interest or astonishment)

Differential criteria:

- Difference from "Adding new meaning"/ "Creating causal links": When the category "Adding new meaning" or "Creating causal links" is chosen, the T's contribution is interpretative. Upon "Drawing attention to a behavioral and/or cognitive pattern" it is more descriptive.

  → In addition, a subcategory of "Interpreting"" might apply.
- Difference from "Repeating, paraphrasing, summarizing": "Drawing attention to a behavioral and/or cognitive pattern" is more than "Repeating paraphrasing, summarizing"; here, the P's utterances are being processed by the T and described in a more abstract manner.
- Furthermore, "Drawing attention to a behavioral and/or cognitive pattern" has to be differentiated from a overall judgment of the whole person (otherwise use category **"**Other")

Example:

T: i just noticed, right? it happens quickly that an inquiry or something - you immediately interpret as criticism or that you feel offended by it this really seems to happen quickly (p:00:00:29) and that inquiring can be helpful - to discover something together? - you cannot imagine that?
 (**"Drawing attention to a behavioral and/or cognitive pattern", ranked as „5"** plus "Adding new meaning", ranked as "5")

3. Highlighting discrepancies

- The T highlights contrasting aspects *or*
- Draws attention to discrepancies or contradictions of thoughts, experiences or behaviors (also with respect to prior sessions).

- Also code this category if it occurs in combination with “Drawing attention to a behavioral and/or cognitive pattern“ or “Adding new meaning”.

Examples:

T: so it seems that you quickly feel hurt or have the feeling another person regards you as being pretty mean
(“Drawing attention to a behavioral and/or cognitive pattern”, ranked as „4”)
P: well because i never! say anything
T: uh-huh
P: never! ----
T: but just then you did say something
(**“Highlighting discrepancies”, ranked as „5”**)

T: one the one hand? you describe yourself as being pretty shy and say “i was always like that?”, i don’t
P: yes but the ones i know!+ (laughs)
T: ,establish contacts? and then? you describe yourself as a rather cheerful! lively! + person.
(**“Highlighting discrepancies”, ranked as „5”** plus “Repeating, paraphrasing, summarizing”, ranked as „3“)

4.x Indicating parallels

The following features apply to the numbers 4.1 to 4.7:

- The T suggests a common denominator between different utterances, topics, experiences of the P.
- Also includes descriptions of experiences from a different time, as long as an element of the common denominator is described.

Exclusion criteria:

- The connection cannot have been made by the P earlier in the session.

4.1 Implicitly indicating a parallel

- The T asks about possible parallels between experiences (e.g., “Does this remind you of something?“).
- The T has to highlight the existence of a parallel but does not have to go into detail about the specific thematic content of the parallel.

Example:

T: i could imagine that this is a situation you! actually know! that you have the feeling your boundaries are being trampled on and someone invades! your space? - where you probably! often hope that your counterpart actually knows! what happens right there with you (P coughs)
(**“Implicitly indicating a parallel”, ranked as „5”** plus “Adding new meaning”, ranked as “3”)

4.2 Parallel without relationship context

- The T indicates parallels between different topics, thoughts, experiences, behaviors.
- The parallels do not refer to a relationship.
- It is sufficient that the T highlights a parallel; it is not necessary that the T states wherein it lies.
- Example: “There goes the topic again…“ would also be allocated to this category.

Differential criteria:

- Difference from “Association“: If a connection/ parallel is established = “Parallel without relationship context“. Without a clear connection/ parallel, e.g., ”This reminds me…” “I have experienced something similar...” = “Association”.

Example:

T: something is being done to me - without me actually being fully in the picture of what it is, without anyone telling me what is going on (p:00:00:07) in principle, this would be very similar! to the state of one week ago, right?
(“Parallel without relationship context”, ranked as “5”)

4.3 Parallel regarding behavior towards the self oneself

- The T indicates parallels between the P’s behavior towards him- or herself and the manner others have behaved towards the P (“You treat yourself like others treat you“).
- The use of a reflexive verb alone does not suffice.

Example:

T: it seems to me as though this is part of you? your mothers opinion, right? well because - you also - offer yourself to others with the eyes of your mother, me included - you are putting yourself down! with respect to what you accomplish here, i have been thinking the whole time, it is incredible that you dare to do this alone and you are putting yourself down - as though your criticizing! mother was inside you!
(“Parallel regarding behavior towards oneself”, ranked as “5” plus “Referring to the therapeutic relationship“, ranked as “4“ plus “Adding new meaning”, ranked as “4” plus “Self-disclosure”, ranked as “3” plus “Expression of emotional sympathy”, ranked as “3”)

4.4 Parallel regarding role reversal

- The T indicates parallels between the P’s behavior towards others and the manner others have behaved towards the P (“Others treat you, like you treat them“).

Example:

T: so if we were going to be more upfront with each other, i would for example uh if i didn’t have to be afraid that you would take it the wrong way, i would have to tell you now that it is outrageous that you are coming here

and saying “i have thought about not coming back again.” just like your mother enters the room and threatens with a farewell! only because something happened in the last session that didn’t suit you, right?
(**“Parallel regarding role reversal”, ranked as “5”** plus “Drawing attention to a behavioral and/or cognitive pattern”, ranked as „2” plus “Self-disclosure”, ranked as “5”)

4.5 Parallel regarding others like parents

- The T establishes an association between the P’s perception of the behavior of one or more current object relations (e.g., partner, therapist) and a significant carer (e.g., mother).
- The T links the past and the present (“Others like my parents“).

Examples:

T: you don’t dare to show me your anger, because you think that i have a violent temper like your father.
(“Parallel regarding others like parents”, ranked as “5”)

T: it seems that the feeling you carry with you from your childhood is “no one is really interested”
(“Parallel regarding others like parents”, ranked as “5”)

4.6 Parallel regarding relationships without linking the past and present

- The T establishes a parallel between general interpersonal behavioral patterns or experiences of interpersonal relationships *or*
- Between the experiences of different current or past interpersonal relationships (concrete examples needed).

Example:

P: i did notice this when i was on the phone to my mum yesterday + and?
T: uh-huh+
P: and maybe it has always been like this
T: and the day before yesterday with your dad you also said that you wanted to know! something and he sort of - kind of made fun of you
(“Parallel regarding relationships without linking the past and present”, ranked as “5”)

4.7 Referring to the therapeutic relationship

- The T brings an utterance into the “here and now” of the therapeutic relationship. The category does not apply if the therapeutic utterance follows an utterance by the P, which already referred to the therapeutic relationship. Exception: Although the therapeutic relationship is already being discussed, the T connects it to the “here and now“ (this had previously not been discussed!). In addition, it is possible that the therapeutic relationship has already been discussed prior in the session and that the T focuses on it again.

- Often (although not necessarily) one of the “Indicating parallels“ categories also applies.
- Also choose this category if the same topic or aspect of the therapeutic relationship is being repeatedly discussed in the here and now.

Exclusion criterion:

- Do not choose this category if the therapeutic relationship is already being discussed.
- Example: P says “I left the session on Monday feeling irritated“, T reacts “It seems like you felt unfairly treated by me“ → The example does not apply to the category, given that the P already made a nonspecific connection to the therapeutic relationship. – By saying “When I left the session” the context of the therapeutic relationship is alluded to.

Example:

P: i i realize a lot of things myself! through a thought-provoking impulse or other things but
T: uh-huh
P: somehow - when i invest in something nothing comes from it
T: uh-huh you experience this between us too - you also wish that more would come from it
(**“Referring to the therapeutic relationship”, ranked as “5”** plus “Parallel regarding relationships without linking the past and present”, ranked as “5”)

5 Exploring

- The T asks about topics that have not been discussed thus far *or*
- Gathers new information without asking a direct question (Example: T: “I am wondering...“, “We could think about…“, “Could you have a closer look at…”).
- Also includes prompts to fantasize.

Example:

T: had you ever suspected this? so, were there ever - hints! that - strengthened! this, so did she - voice(p:00:00:08) that she only values the smart friends
(**“Exploring”, ranked as “5”**)

6.x Interpreting

The following features apply to the numbers 6.1 to 6.3:

- The T interprets the utterances of the P *or*
- The T offers something new (own ideas).
- What is offered by the T (i.e., the interpretative effort) has to go beyond a repetition or differentiated summary of prior contents (which can also lead to new connections and complex new meanings).
- The offer of the T (e.g. own idea) can be expressed as a question.

Exclusion criterion:

- The idea cannot have been previously expressed by the P.

6.1 Adding new meaning

In response to the content described by the P, the T tries to:

- Interpret the content, add new meaning to it *or*
- Link the content to a context not previously verbalized by the P *or*
- Integrate the content into a new context *or*
- Introduce a new perspective. The T completes/ adds something to the P's utterances. Caution: it has to be derived from the P's utterances. The T refers primarily to the P's self, offers information that is more congruent with the P's self, when compared to "Suggestion". For example "Maybe your mother wanted to assume the role of a girlfriend" would be a possible new perspective on a mother who has been described as liberal, while "Imagine your mother as your girlfriend" describes a "Suggestion" which is less P-congruent.
- Also choose this category, if the T offers the same meaning multiple times in the course of a session. In other words, the added meaning is not "new" anymore. "New" means that the therapeutic utterance goes beyond what the P said, that it contains interpretations from T.

Differential criteria:

- Difference from „Drawing attention to a behavioral and/or cognitive pattern": "Adding new meaning" is chosen when T's therapeutic utterances are more interpretative; „Drawing attention to a behavioral and/or cognitive pattern" is chosen when T's therapeutic utterances are more descriptive.

Example:

P: well maybe it's (p:00:00:09) --- maybe it's like - being messed! with or like being left in the dark
T: uh-huh
P: not noticing anything
T: so it's anger! the tears
(**"Adding new meaning", ranked as "5"**)

6.2 Creating causal links

- The T makes causal links between thoughts, feelings or behaviors.
- The focus is on causality (e.g., "because…"), not a connection.

Example:

T: well maybe you sort of did it yourself due to - the fact - that you somehow - feel shame!

P: +uh-huh
T: maybe+ that’s how it is!
(**“Creating causal links”, ranked as “5”** plus “Adding new meaning”, ranked as “5”)

6.3 Interpretation using metaphors

- The T introduces an allegory, narrative, proverb or aphorism to make the P’s *complex* experience more tangible and/or to establish connections.
- The metaphor has to offer new input. It has to add meaning, which would not have emerged without the metaphor.

Exclusion criterion:

- It goes beyond pure repetition and a summary in figurative language!

Example:

T: here i am also! not different to your parents - where only - the reputation of the family counts and the belief and the community - +how can
P: yes and+
T: how can you so to speak leave the church
(**“Interpretation using metaphors”, ranked as “5”** plus “Adding new meaning”, ranked as “5” plus “Referring to the therapeutic relationship”, ranked as “5” plus ““Parallel regarding others like parents”, ranked as “5”)

7 Encouraging a view or impulse

- The T encourages an impulse, thought, view or behavior that the P had not dared to openly discuss. The P has to have previously hinted at this (otherwise “Suggestion“ probably applies).
- The T encourages a way of thinking or a viewpoint *or*
- Pushes a course of action.
- The T’s input is based on the experience of the P.

Exclusion criterion:

- Do not choose this category if the T disagrees with the P or deviates from the experience of the P.

Example:

P: i have to add of course! that i am not necessarily very outgoing! but i never was -
T: are you - sort of saying “this is! how i am? i will stay! like this?” or are you saying “it’s actually a shame” and so who knows! maybe i would make new experiences!? +with other people”
(**“Encouraging a view or impulse”, ranked as “5”**)

8 Validation

- The T expresses encouragement, validates a view, perception, behavior or impulse of the P.
- Input of the T is based on the experience of the P.

Exclusion criterion:

- Do not choose this category if the T disagrees with the P or deviates from the experience of the P.

Example:

P: so then i say to myself "let him be?" and - "i don't have! to talk to him" so
T: uh-huh of course you don't have to
(**"Validation", ranked as "5"**)

9 Suggestion

- The T offers something he/she likes, which is "foreign" to the P's self.
- The input of the T is *not* directly based on the experience of the P.
- The T gives recommendations, advice *or*
- Proposes solutions and his/her experience *or*
- Asks the P to do or to refrain from doing something.
- Clear instruction to think or act differently/ in a new manner (This is in contrast to "Drawing attention to a behavioral and/or cognitive pattern", where the T merely describes).

Differential criteria:

- Do **not** choose this category if therapeutic utterances merely contain implicit suggestions, e.g. "You cannot be appalled."
- Example: "You cannot imagine that I want to do something good for you" is not a "Suggestion" if it is derived from the experience of the P. Here, it needs to be checked, whether the category "Drawing attention to a behavioral and/or cognitive pattern" or a subcategory of "Interpreting" applies.
- Example: "One could be appalled at this point." or "You could actually be appalled here" would apply more to "Adding new meaning" (depending on the context, of course). A "Suggestion" could sound like this: "You should just be appalled at this point".

Example:

T: uh-huh - maybe with this +training program you can already head in that direction!?
(**"Suggestion", ranked as "5"**)

10 Self-disclosure

- The T shares his/ her personal experience with the P.

- The T communicates the effect the P's utterances and behaviors have on the T; e.g., which feelings and reactions they trigger in the T (positive and negative).
- The T describes his/ her own emotional reactions (Example: "I feel helpless when…") or intentions ("I said this to…"; "I did not want to accuse you…"; "I am trying to think about whether you were right about…"; "I cannot understand you when you react like this…").
- A therapeutic utterance does not belong to this category merely because the T says the word "I".
- Always code Dimension 2 as "T".

Differential criteria:

- Do not choose this category for generic therapeutic utterances.
- Expressing interest or astonishment is not self-disclosure. Instead, it usually is an introductory therapeutic utterance, which is followed by utterances that belong other categories → Exception: The explicit focus of the therapeutic utterance lies within the T expressing his/ her interest or astonishment.

Example:

T: uh-huh --- yes you probably somehow learned this right? that - your sadness - or such feelings - make you helpless! and that this is something you should be ashamed! of and that you are kind of cornering people but this - um --- well i don't feel cornered by it
(**"Self-disclosure", ranked as "5"** plus "Adding new meaning", ranked as "3")

11 Association

- The T recalls thematic content which was mentioned by the P in previous sessions *or*
- The T reveals pictures, memories, stories etc. which occur to him or her.

Differential criteria:

- Contrast to "Parallel without relationship context": A connection/ similarity is established and the focus is on the parallel = "Parallel without relationship context". Without a clear connection/ parallel „This reminds me of…" = "Association".

Examples:

T: so when i think of the childhood story with the truck driver, right?
(**"Association", ranked as "5"**)

T: this reminds me of the dream you told a few sessions ago
(**"Association", ranked as "5"**)

12 Expression of emotional sympathy

- Verbally expressed reaction to the P’s utterances (sympathy, comfort, emotional consolation with the P’s experience).
- Input of the T is based on the experience of the P.

Differential criteria:

- Do **not** choose this category if the therapeutic utterance only expresses implicit emotional consolation with the P’s experience.
- “I want to do good by you“ would fit more to the category “Self-disclosure“ (The intention of the T is more apparent).

Example:

T: uh-huh (p:00:00:10) that is hard
(“Expression of emotional sympathy”, ranked as “5”)

13 Conveying professional knowledge

- The T conveys professional knowledge, explains an issue or a fact, remains abstract, general, theoretical.
- The T remains neutral (does not offer personal opinion or tendency).

Example:

T: there is this mechanism whereby children protect themselves by - trying to empathize! -- with others, with the adult they cannot understand! - it cannot be comprehended what your father did to you, but as i said, children sometimes attempt? it by reenacting it! doing a role play with other children for example - or they try to empathize using their imagination! to reach some! kind of understanding which cannot be reached!
(**“Conveying professional knowledge”, ranked as “5”** plus “Creating causal links”, ranked as “5” plus “Parallel regarding role reversal”, ranked as “2”)

14 Other

- All utterances, which do not fulfill the criteria of the other categories.
- Attention! Do not neglect this category!

Examples

- Organizational matters; Gathering factual information.
- Utterances that deviate from the regular process, e.g., utterances concerning the beginning or end of a session (Attention: utterances such as “Let us continue on Monday“ can also serve to end a session).
- Special cases, e.g., when the T completes a sentence that was started by the P.
- Features of utterances that do not (completely) apply to any category (can also concern parts of utterances – in this case choose “Others“ as an additional category).

Example:

T: Have a nice weekend.
(**“Other”, ranked as “5”**)

15 Single filler words

- Do not combine filler words with other categories!

.1 Single filler words → Examples: "oh well“ "humph“ "uh-huh“ "aha“ "sure“ "I see“ "well…“

.2 Sentence fragments (i.e. therapeutic utterances) which are not completed in the following therapeutic utterance and do not form a complete sentence → Example: This would apply to “What a“ and also “yes but“ because a further therapeutic utterance is expected/ the therapeutic utterance cannot stand alone.

- Do not choose .1 and .2 simultaneously. Either .1 or .2

Dimensions 2 and 3 – Thematic content (9 categories) and temporal focus (4 categories)

The categories are used to rate formal features of therapeutic utterances. The dimensions 2 and 3 are used to rate what the utterances refer to. They describe the thematic content and temporal focus of the conversation at the time of an utterance.

Filler words should also be allocated dimensions (usually, the categories can be deduced from the previous utterance of the P or the T).

The dimensions 2 and 3 are usually pretty clear. Occasionally, a clear allocation is not possible, e.g., if the thematic focus shifts between the therapeutic relationship (T) and abstract relationship behavior (K); Or if the symptomatology (S) and the abstract experience of the P (K) are discussed simultaneously. In this case, both (several) categories have to be assigned.

Dimension 2 (Thematic content)

T = Therapist

- The therapeutic relationship is being discussed.

CO = Current object relation

- Current relationships are being discussed (e.g., partner, children, friends, colleagues etc.; fellow patients are also allocated to this dimension).
- Only code “A“ if a specific relationship is being discussed. If a more abstract relationship is being described (i.e., “The people at the wedding”), choose reference “K”.

PO… = Primary object relation

- Significant others of the P‘s childhood and adolescence (e.g., parents, siblings) who have shaped the personality structure.
- Also code this if current primary object relations are being discussed.

POM = Mother

POV = Father

POX = Unspecified primary object relation

POY = Other primary object relations

A = Abstract relationship behavior

- General (abstract) relationship behavior without a specific reference person.
- Only choose this category if no specific person is being discussed (Example: ”I don’t get along with some people.” The utterance “The people at the wedding…” also has to be allocated to “K”, as no specific person is being described.)
- Do not code “K“ in addition to other categories simply because ”something abstract is always being discussed”.

S = Symptomatology

- The symptoms of the P are being discussed.
- This exclusively refers to physical or psychological symptoms (e.g., panic attacks, claustrophobia, depression, compulsions, eating disorders, breathing difficulties, pain, itching, colds, sleep problems…). Not: interpersonal problems.

X = Content without relationship- or symptomatology-context

- Other references do not fit.
- The P says ”I feel best when I can sit at home and read books” →Any relationship context is implicit and can only be assumed. Therefore code “X”. (If the P would say: ”When there are lots of people around me/ When others come too close to me, it makes me feel unwell. Sometimes I therefore like it best to be home alone and to read.” – rather choose “K”.)
- For general content, e.g., organizational matters.
- This category also applies to utterances that exclusively refer to the beginning or end of a session, unless they explicitly refer to a different category.

Different categories are to be assigned simultaneously if they all apply, e.g., categories S and T if symptoms and the therapeutic relationship are being discussed simultaneously.

Dimension 3 (Temporal focus)

P = Presence

- Inner experiences, ideas or seemingly unrealistic thoughts (e.g., fears, worries, concerns), even if they do not appear to be substantiated by actual experiences. Example: “What would happen if you followed your impulse“, “How would your boss react if you did speak your mind?”
- Present and past, unless the childhood or adolescence of the P is being discussed.
- Utterances that refer to the beginning or end of a session (Exception: There is a clear reference to a different temporal focus).

C/A = Childhood/ Adolescence

- The childhood or adolescence of the P is being discussed.

Sy = Symbol

- A dream, metaphor or fairytale is being discussed. (Only choose this if a topic is addressed by means of a dream/ picture/ fairytale. Do not automatically code this if the T uses figurative language or engages in a metaphorical interpretation.)

X = Other temporal foci

- Other categories to do fit.

Different categories can be allocated simultaneously, if they all apply e.g., categories Sy and P or Sy and C/A.
